# Ureaplasma parvum meningitis in neonates: A retrospective case study and literature review

**DOI:** 10.12669/pjms.42.8.16496

**Published:** 2026-08

**Authors:** Fang Yan, Meifang Lin, Qinqin Fu, Yi Gao, Yeting Hu, Minfen Zhou

**Affiliations:** 1 Fang Yan Department of Pediatrics, Huzhou Maternity & Child Health Care Hospital, Huzhou, Zhejiang Province 313000, P.R. China; 2 Meifang Lin Department of Pediatrics, Huzhou Maternity & Child Health Care Hospital, Huzhou, Zhejiang Province 313000, P.R. China; 3 Qinqin Fu Department of Neonatology, Huzhou Maternity & Child Health Care Hospital, Huzhou, Zhejiang Province 313000, P.R. China; 4 Yi Gao Department of Pediatrics, Huzhou Maternity & Child Health Care Hospital, Huzhou, Zhejiang Province 313000, P.R. China; 5 Yeting Hu Department of Pediatrics, Huzhou Maternity & Child Health Care Hospital, Huzhou, Zhejiang Province 313000, P.R. China; 6 Minfen Zhou Department of Neonatology, Huzhou Maternity & Child Health Care Hospital, Huzhou, Zhejiang Province 313000, P.R. China

**Keywords:** Meningitis, Neonate, Ureaplasma, Ureaplasma parvum

## Abstract

**Objective:**

To investigate the clinical characteristics and advances in diagnosis and treatment of neonatal *Ureaplasma parvum* (*U. parvum*) meningitis.

**Methodology:**

Clinical manifestations, diagnosis, treatment, and follow-up data of a neonate with persistent fever, normal blood routine, but persistently abnormal cerebrospinal fluid (CSF) results due to *U. parvum* meningitis were retrospectively analyzed. A literature review was conducted to identify relevant studies reporting on neonatal *U. parvum* meningitis published until May 2025.

**Results:**

A male infant born at 35+2 weeks of gestation with eight hours of premature rupture of membranes (PROM) was admitted at 17 days of age with persistent fever. The routine blood test was normal, while the CSF suggested purulent meningitis. Empirical treatment was ineffective, and metagenomic next-generation sequencing (mNGS) of CSF confirmed *U. parvum* meningitis. The infant recovered after three weeks of azithromycin treatment, but a language delay was found at two-year follow-up. The literature review identified 16 previously reported cases of *U. parvum* meningitis, which were combined with the current case, totaling 17 cases. Main manifestations included fever and convulsions, or initial circulatory and respiratory symptoms. CSF in the included studies showed leukocytosis, decreased glucose, and elevated protein. Diagnosis was mainly based on mNGS, and the predominant treatment consisted of macrolides, sometimes combined with quinolones, for 3–10 weeks. Two patients relapsed after drug withdrawal, and one had elevated liver enzymes. Major neurological complications included lateral ventricular dilatation and hydrocephalus, cerebral hemorrhage, infarction, malacia, and herniation. Most cases had a favorable prognosis, with rare developmental delay.

**Conclusion:**

Clinical manifestations of neonatal *U. parvum* meningitis are nonspecific. Some patients present with normal blood routine but purulent CSF. *U. parvum* meningitis should be suspected in patients with poor response to empirical antibiotics and confirmed by CSF mNGS. Macrolides are biologically rational and commonly used for neonatal *U. parvum* meningitis; however, the optimal regimen and duration remain unclear due to limited and heterogeneous case-based evidence.

## INTRODUCTION

Ureaplasma species (*Ureaplasma spp*.), including *Ureaplasma parvum (U. parvum)* and *Ureaplasma urealyticum (U. urealyticum)*, are recognized as etiological agents of adverse neonatal outcomes, including congenital pneumonia, bronchopulmonary dysplasia, and perinatal death.^1^,^2^ However, neonatal meningitis caused by invasive *U. parvum* infection is rarely reported and remains inadequately characterized in the literature.^3^,^4^ Currently, most reports of neonatal meningitis caused by *U. parvum* infection are sporadic case reports and lack a systematic summary. This study aimed to present a representative case and perform a comprehensive review of both Chinese and international literature, systematically summarizing the clinical features, diagnostic and therapeutic approaches, and prognosis of *U. parvum* meningitis, to provide more reliable evidence for early recognition and standardized management.

## LITERATURE REVIEW

A systematic literature search was performed in PubMed, Embase, Web of Science, and CNKI from database inception to May 2025. The following search terms were used: (“Ureaplasma parvum” OR “U. parvum”) AND (“meningitis” OR “central nervous system infection”) AND (“neonate” OR “newborn” OR “infant”). No language restrictions were applied, and both English and Chinese articles were included.

## CASE INCLUSION CRITERIA

### Cases were included in this review if they met all of the following criteria:

1. Infants diagnosed with meningitis caused by U. parvum
2. The causative pathogen was confirmed by species-level identification (e.g., species-specific PCR, mNGS, or culture with subsequent species identification as U. parvum)
3. The diagnosis of meningitis was supported by clinical signs (e.g., fever, irritability, seizures, or altered consciousness) and/or consistent cerebrospinal fluid (CSF) findings (pleocytosis, elevated protein, and/or decreased glucose)
4. Cases reported only as *Ureaplasma spp*. without species-level differentiation were excluded
5. Cases involving U. urealyticum or mixed bacterial/viral infections were excluded.

## CASE PRESENTATION

The patient was a 17 days old male infant, the second child of a third pregnancy, delivered at 35+2 weeks of gestation with a birth weight of 2520 g. Premature rupture of membranes (PROM) occurred eight hours prior to delivery, with clear and odorless amniotic fluid. A maternal cervical secretion culture was positive for *Mycoplasma* spp. Following birth, the infant was admitted to the Neonatal Intensive Care Unit (NICU) due to neonatal respiratory distress syndrome and prematurity, receiving invasive mechanical ventilation, intratracheal surfactant administration, and parenteral nutrition for 14 days before discharge. Three days after discharge, the infant was readmitted with a fever lasting more than one hour.

### Ethical approval:

The ethics committee of our hospital approved this case report with the number: 2025-J-067, Date: August 28, 2025.

Upon admission, the infant was empirically treated with piperacillin-tazobactam (100 mg/kg per dose every eight hours). On the second day, the cerebrospinal fluid (CSF) analysis revealed a leukocyte count of 81×10^6^/L, protein concentration of 1.4 g/L, and glucose level of 2.0 mmol/L, leading to a clinical diagnosis of neonatal purulent meningitis. Consequently, the antibiotic regimen was adjusted to ceftriaxone (50 mg/kg per dose every 12 hours). Although serial complete blood counts remained within normal limits and both blood and sputum cultures showed no bacterial growth following admission, a repeated CSF analysis on day 11 demonstrated pleocytosis (220×10^6^/L), markedly elevated protein levels (1.80 g/L), and significantly decreased glucose concentration (1.5 mmol/L), indicating an inadequate response to the initial antibiotic treatment. Accordingly, the antimicrobial therapy was adjusted to meropenem, administered intravenously at a dosage of 40 mg/kg every eight hours. On day 12, cranial magnetic resonance imaging (MRI) revealed widening of the left extra-axial space, cerebral sulci, and the left interhemispheric fissure, with localized meningeal thickening, findings suggestive of purulent meningitis.

**Fig.1:**
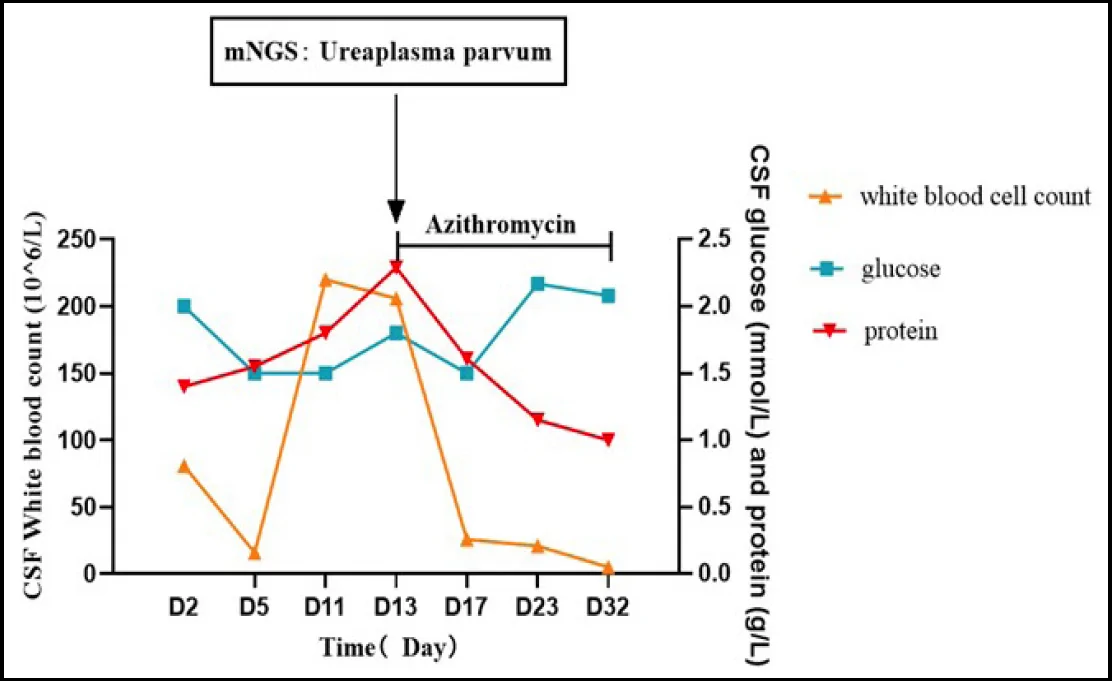
The temporal variations in cerebrospinal fluid white blood cell count, protein concentration, and glucose levels.

On day 13 of hospitalization, mNGS of CSF was performed. The assay detected 27 unique reads specifically mapped to *Ureaplasma parvum* with a confidence level of 99% and a relative abundance of 69.3%. No reads of *Ureaplasma urealyticum*, bacteria, fungi, or viruses were identified, and the sequencing quality was adequate. Accordingly, antibiotic therapy was switched to azithromycin. Subsequent CSF analyses demonstrated progressive improvement in routine and biochemical parameters (Fig.1), and follow-up MRI revealed a reduction in the extra-axial space. Azithromycin was administered over a three-week period, with a dosing regimen of 20 mg/kg daily during the initial two weeks, followed by 10 mg/kg daily for the final week. Throughout the treatment period, complete blood counts, liver function tests, electrocardiograms, and pyloric ultrasound findings all remained within normal limits. At follow-up, post-discharge cranial MRI showed complete resolution of meningeal thickening and normalization of the extra-axial space. However, at the two-year follow-up, the infant was noted to have language developmental delay.

## DISCUSSION

This case study presents a preterm infant with a history of PROM, a known risk factor for perinatal infection.^5^ The patient presented with fever as the primary clinical manifestation. Investigations ruled out underlying cellular or humoral immunodeficiencies, as well as infections with enterovirus, cytomegalovirus, and fungi. It is noteworthy that throughout the course of the disease, the infant repeatedly showed normal routine infection markers, including complete blood count (with granulocyte count), C-reactive protein, and procalcitonin, while CSF analysis consistently indicated clear evidence of central nervous system infection (elevated white blood cells, increased protein, decreased glucose). This discrepancy between normal blood tests and abnormal CSF, along with the poor response to multiple antibiotics initially administered for purulent meningitis, suggested an uncommon pathogen causing the neurological infection. mNGS of the CSF identified *U. parvum*, while a culture of the mother’s cervical secretions concurrently isolated *Mycoplasma spp*, leading to the diagnosis of *U. parvum* meningitis. The infant exhibited clinical improvement following a three-week course of azithromycin.

While there has been an increase in reported cases of neonatal *U. parvum* meningitis in recent years, clinical understanding of this condition remains limited. *U. parvum* primarily colonizes the human urinary and reproductive tracts, with infection rates in the female reproductive tract ranging from 40%-80%.^6^ Such colonization is associated with adverse pregnancy outcomes, including chorioamnionitis, preterm birth, and premature rupture of membranes.^7^,^8^ Furthermore, *U. parvum* colonization may lead to intrauterine fetal infection or neonatal acquisition during delivery, potentially resulting in respiratory, gastrointestinal, and central nervous system complications.^9^

A total of seven Chinese-language articles^10^-^16^ and eight English-language articles^17^-^24^ were reviewed, identifying 16 previously reported cases. Together with the present case, a total of 17 cases were included in this review. Demographic characteristics, diagnostic and treatment parameters, and clinical outcomes of the patients are summarized in Tables-I-III, respectively. Neonatal *U. parvum* meningitis often presents with insidious fever and may be accompanied by seizures. In some cases, it manifests initially with circulatory or respiratory symptoms such as cyanosis, tachypnea, and apnea. Occasional cases may involve metabolic disturbances, including hyperglycemia. The primary neurological complications include lateral ventricular dilatation and hydrocephalus, and cerebral hemorrhage.^25^

**Table-I:** Demographic and clinical characteristics (N=17).

Case No.	Gender	Gestation (week)	Birth weight (g)	Symptom
1	Male	39+5	3600	Fever^10^
2	Male	35+6	2980	Fever, tachypnea^11^
3	Female	31+6	1600	Fever, lethargy^11^
4	Female	41+1	3940	Fever, seizures^12^
5	Female	29	1350	Poor response, apnea, cardiovascular instability^13^
6	Male	39+6	3250	Fever, hyperglycemia, seizures^15^
7	Female	27	1080	Tachypnea^16^
8	Female	30+2	1450	Tachypnea, cyanosis, seizures^16^
9	Male	26	979	Tachypnea^16^
10	Not Reported	39	3500	Fever, conjunctivitis, seizures^18^
11	Male	Full term	Not Reported	Fever, irritability, seizures^19^
12	Male	40	3390	Fever, seizures hypotonia^20^
13	Male	40	3800	Fever, grunting tachypnea, seizures^21^
14	Female	30	1630	Fever, apnea, cardiovascular instability^22^
15	Female	26+3	940	Poor response, hypotonia^20^
16	Female	28	950	Fever, cyanosis, tachypnea^24^
17	Male	35+2	2520	Fever

**Table-II:** Diagnostic and therapeutic methods (N=17).

Case No.	Test method	Treatment
1	mNGS	Azithromycin (10→5 mg/kg/d, total 35d)^10^
2	mNGS+PCR	Erythromycin×4d (Ineffective) → Azithromycin×4w^11^
3	mNGS+PCR	Azithromycin×3w^11^
4	mNGS	Azithromycin (10 mg/kg/d)×4w^12^
5	mNGS	Erythromycin×3w^13^
6	mNGS+PCR	Azithromycin×1w → Erythromycin×1w (Ineffective) → Azithromycin×3w^15^
7	mNGS	Azithromycin (20 mg/kg/d)×3w^16^
8	mNGS	Ciprofloxacin×1w → Levofloxacin×2w → Moxifloxacin×7w^16^
9	mNGS+PCR	Erythromycin×32d^17^
10	PCR+culture	Ciprofloxacin×7w + Thiamphenicol×3w^18^
11	PCR	Erythromycin+Ciprofloxacin×5w → Azithromycin+Ciprofloxacin×1w^19^
12	mNGS	Erythromycin (10 mg/kg/dose q6h)×4w^20^
13	mNGS+culture	Erythromycin×5w^21^
14	mNGS+culture	Erythromycin+Ciprofloxacin×3w^22^
15	mNGS+culture	Chloramphenicol×3w^23^
16	mNGS	Erythromycin (30 mg/kg/d)+Ciprofloxacin (20 mg/kg/d)×3w^24^
17	mNGS	Azithromycin×3w

***Abbreviations:*** PCR- Polymerase chain reaction; mNGS- Metagenomic next-generation sequencing.

**Table-III:** Neurological complications and outcomes (N=17).

Case No.	Complication	Prognosis
1	Hydrocephalus	Global developmental delay^10^
2	Lateral ventricular dilatation, Hydrocephalus	Normal (9,14 months)^11^
3	Lateral ventricular dilatation	Normal (18 months)^11^
4	Hydrocephalus, intraparenchymal hemorrhage	Normal (36 months)^12^
5	Lateral ventricular dilatation, Hydrocephalus	Normal (12 months)^13^
6	ICH,SAH Hemorrhagic infarction, Cerebral herniation	Motor and language delay^15^
7	Lateral ventricular dilatation, encephalomalacia Hydrocephalus, ICH,	Normal (12 months)^16^
8	ICH, Lateral ventricular dilatation, Hydrocephalus	Cerebral palsy (24 months)^16^
9	Lateral ventricular dilatation, Hydrocephalus ICH,SEH	Normal (5 months)^17^
10	Lateral ventricular dilatation	Normal (8,14 months)^18^
11	Lateral ventricular dilatation	Normal (30 months)^19^
12	Lateral ventricular dilatation	Normal (18 months)^20^
13	Subdural hemorrhage; Cerebral atrophy	Normal (5 months)^21^
14	ICH, Hydrocephalus	Normal (6 months)^22^
15	Hydrocephalus	Normal (12 months)^23^
16	Lateral ventricular dilatation, Hydrocephalus	Not Reported^24^
17	Extra-axial space enlargement Meningeal thickening, SAH	Language delay

***Abbreviations:*** ICH- Intraventricular hemorrhage; SAH- Subarachnoid hemorrhage; SEH- Subependymal hemorrhage.

The CSF profile is typically characterized by an elevated white blood cell count, increased protein levels, and decreased glucose concentration, resulting in purulent changes that are difficult to distinguish from bacterial neonatal meningitis. Notably, CSF glucose levels in *U. parvum* meningitis are often significantly lower, sometimes undetectable, and CSF cultures are generally negative.^26^,^27^ Conventional laboratory diagnosis of *Ureaplasma* infection has traditionally relied on culture methods and polymerase chain reaction (PCR), which present certain limitations. In contrast, mNGS facilitates the early diagnosis and precise treatment of *U. parvum* meningitis by enabling rapid, accurate pathogen detection, thereby significantly improving patient prognosis.^27^

Currently, there is no consensus on the optimal antimicrobial regimen or treatment duration for neonatal *U. parvum* meningitis. Macrolides are clinically preferred and biologically rational, as *Ureaplasma* species lack a cell wall.^28^ Fluoroquinolone combination therapy is occasionally administered for severe cases, but supporting evidence is limited to isolated case reports with substantial variability in treatment protocols, durations, and clinical outcomes. No randomized controlled trials have validated the superiority of any specific regimen or duration, underscoring the urgent need for standardized data from further investigations.^29^ Moreover, for cases complicated by severe intraventricular hemorrhage (Grade-III or above) or progressive hydrocephalus, a combined approach involving neurosurgical intervention, such as ventriculoperitoneal shunt placement or reservoir implantation, alongside pharmacotherapy is recommended.^14^,^18^ Although most neonates with *U. parvum* meningitis experience favorable outcomes, including complete resolution of neurological symptoms in some cases, a subset may develop severe sequelae, such as obstructive hydrocephalus, neurodevelopmental delays, or cerebral palsy.^15^,^17^ Owing to the heterogeneous assessment methods used by the original study authors—including standardized tools (e.g., Ages & Stages Questionnaires, Denver Developmental Screening Test), routine clinical examinations by pediatricians, and parent-reported developmental milestones—and variable follow-up durations ranging from five to 36 months, the interpretation of long-term prognosis should be made with caution.

*U. parvum* meningitis is clinically rare, and current understanding of this disease derives mainly from isolated case reports. Through a systematic literature search, this study comprehensively analyzes 16 previously reported cases together with the present case, totaling 17 cases, which helps to clarify the clinical features of the disease. The included cases show that affected infants frequently present with low birth weight, vaginal delivery, premature rupture of membranes, and maternal *Ureaplasma* spp. infection during pregnancy. In the absence of a control group, these represent common clinical features rather than confirmed risk factors. Compared with previously reported extremely low birth weight infants with severe multi-system involvement, the present case is a moderate preterm infant with isolated central nervous system infection, without severe perinatal complications such as intraventricular hemorrhage or hypoxic-ischemic encephalopathy. Notably, the infant`s mother had a positive cervical *Ureaplasma* spp. culture, providing clear evidence of maternal-neonatal transmission. Unlike most severe cases requiring combined therapy with erythromycin and ciprofloxacin, this patient achieved a favorable response to azithromycin monotherapy. At two years of age, neurodevelopmental evaluation using the Developmental Screening Test (DST) for Chinese children aged 0-6 years confirmed language delay, with normal gross motor, fine motor, and social-adaptive development.

Notably, *Ureaplasma parvum* and *Ureaplasma urealyticum* exhibit significant differences in biological characteristics, pathogenicity, and clinical phenotypes. However, in clinical practice, they are often collectively referred to as *Ureaplasma* spp., which may lead to the neglect of these critical distinctions. In neonates, *U. urealyticum* causes significant respiratory damage, frequently manifesting as respiratory distress, apnea, and an increased need for respiratory support.^30^,^31^ The pathogenesis of *U. urealyticum* may involve pulmonary colonization and subsequent local inflammation.^30^ In contrast, *U. parvum* infection is often asymptomatic or presents with temperature abnormalities, such as unexplained fever or thermal instability; the initial respiratory symptoms are often atypical and can be easily attributed to prematurity-related complications.^9^ The optimal diagnostic approaches for these pathogens also differ. Given its fastidious culture requirements and low positivity rate, *U. parvum* infection is best identified using cerebrospinal fluid mNGS.^17^ In contrast, *U. urealyticum* infection is best detected using a combination of improved culture methods and specific PCR.^32^ This approach balances timeliness with increased detection sensitivity, facilitating strain typing and surveillance of drug resistance. Genomic studies have shown that pathogenic *U. urealyticum* strains have larger genomes and harbor more complex resistance gene clusters, including those conferring resistance to macrolides and tetracyclines.^33^ This leads to significantly higher clinical resistance rates compared to those of *U. parvum* strains and an increased risk of initial treatment failure.^34^

In contrast, the *U. parvum* genome is relatively compact and remains highly susceptible to macrolide antibiotics.^33^,^35^ Notably, infants with *U. urealyticum* meningitis are more prone to severe complications such as lateral ventricular dilatation, hydrocephalus, white matter injury, intraventricular hemorrhage, and subsequent neurodevelopmental delays and motor dysfunction.^36^ Therefore, for neonates with *U. urealyticum* infection, it is essential to establish and implement a multidisciplinary system for long-term neurological evaluation and rehabilitative intervention to improve the long-term prognosis.^15^,^37^

### Limitations:

First, it is a single-case report, and the findings cannot be generalized to all infants with *U. parvum* meningitis. Second, the literature review may be subject to publication bias, as positive cases are more likely to be reported than negative or mild cases. Third, some clinical data, including follow-up information, are incomplete. Fourth, heterogeneous diagnostic methods (culture, PCR, mNGS) and non-standardized antimicrobial regimens were used across the included cases. Finally, variable follow-up durations limit the evaluation of long-term neurodevelopmental outcomes.

## CONCLUSION

Our findings indicate that clinicians should maintain a high suspicion of *U. parvum* infection in neonates who present with purulent CSF, negative routine cultures and inadequate response to β-lactam antibiotics, particularly in cases of CSF-blood dissociation. In such cases, early mNGS is warranted to enable timely diagnosis. Notably, *U. parvum* can cause both acute meningitis in preterm infants and delayed-onset meningitis in full-term neonates. Given the limited and heterogeneous evidence currently available, definitive recommendations for optimal treatment regimens or risk stratification strategies cannot be provided. Further large-scale, prospective studies are urgently needed to establish standardized diagnostic and therapeutic guidelines for neonatal *Ureaplasma* meningitis.

### Recommendations:

Future multicenter prospective studies are warranted to validate these findings.
